# Mastering your fellowship

**DOI:** 10.4102/safp.v63i1.5282

**Published:** 2021-03-10

**Authors:** Klaus von Pressentin, Mergan Naidoo, Frederick Mayanja, Tasleem Ras

**Affiliations:** 1 School of Public Health and Family Medicine, Faculty of Health Sciences, University of Cape Town, Cape Town, South Africa; 2 Department of Family Medicine, University of KwaZulu-Natal, Durban, South Africa; 3 Department of Family Medicine, Walter Sisulu University, Mthatha, South Africa

**Keywords:** Fellowship of the College of Family Physicians SA examination, family medicine registrars, general adult medicine, critical appraisal, clinical governance

## Abstract

The series, ‘Mastering your Fellowship’, provides examples of the question format encountered in the written and clinical examinations, Part A of the Fellowship of the College of Family Physicians of South Africa (FCFP, SA) examination. The series is aimed at helping family medicine registrars (and their supervisors) prepare for this examination. Model answers are available online.

## Introduction

This section in the *South African Family Practice* journal is aimed at helping registrars prepare for the Fellowship of the College of Family Physician of South Africa (FCFP SA) Final Part A examination and will provide examples of the question formats encountered in the written examination: multiple choice questions (MCQs) in the form of single best answer (SBA – Type A) and/or extended matching question (EMQ – Type R); short answer questions (SAQs); questions based on the critical reading of a journal article (evidence-based medicine); and an example of an objectively structured clinical examination (OSCE) question. Each of these question types is presented based on the College of Family Physicians blueprint and the key learning outcomes of the FCFP programme. The MCQs will be based on the 10 clinical domains of family medicine, the MEQs will be aligned with the five national unit standards and the critical reading section will include evidence-based medicine and primary care research methods.

This month’s edition is based on unit Standard 1 (lead clinical governance activities by facilitating reflection on health information to improve quality of clinical care; facilitating risk management processes and improving patient safety; and critically reviewing new evidence and applying the evidence in practice) and unit Standard 2 (evaluate and manage a patient according to the bio-psycho-social approach). The theme for this edition is general adult medicine. We suggest that you attempt answering the questions (by yourself or with peers and/or supervisors), before finding the model answers online: http://www.safpj.co.za/.

Please visit the Colleges of Medicine website for guidelines on the Fellowship examination: https://www.cmsa.co.za/view_exam.aspx?QualificationID=9.

We are keen to hear about how this series is assisting registrars and their supervisors in preparing for the FCFP SA examination. Please email us your feedback and suggestions.

## Multiple choice question (MCQ) – Single best answer

A 45-year-old male, identified with type 2 diabetes mellitus and hypertension, presents with cough, fever and body pains for 2 days. His blood pressure = 140/88 mmHg, pulse = 100/min, respiratory rate = 22/min, saturation in room air is 96%, blood sugar is 8 mmol/L and his temperature is 37.7 °C. The rest of the examination is unremarkable and the Rapid Antigen Test for severe acute respiratory syndrome coronavirus 2 (SARS-CoV-2) is positive. He is able to self-isolate safely at home. What would be the most appropriate therapeutic intervention at this stage?

- Ibuprofen
- Ivermectin
- Paracetamol
- Steroids
- Vitamins

*Answer*: c)

This is a common scenario facing primary care providers around the world today. The coronavirus disease 2019 (COVID-19) pandemic has had devastating effects on populations, and the new variants seem to be more infectious. This case illustrates a patient with mild disease, but with significant co-morbidities. The focus of the consultation should be on counselling and education, focussing on measures needed at home to prevent others at home from being infected. The patient should also be counselled on the symptoms and when to seek immediate medical attention. Most patients who deteriorate develop dyspnoea within 5 days of symptom onset and required hospitalisation. Any deterioration in the ability to perform activities of daily living at home should prompt immediate re-evaluation. Because of the comorbidities it would be prudent to re-evaluate this patient at day 6 to establish if the disease has progressed. Some primary care clinicians measure markers (such as a C-reactive protein and/or D-dimer) to assist them with their clinical decision-making process. When resources permit, other useful measurements of inflammatory markers include Interleukin 6 and ferritin levels as well as the neutrophil-to-lymphocyte ratios. The neutrophil-to-lymphocyte ratio, when elevated, may suggest that a patient is at higher risk of developing severe disease or death. It is sometimes very difficult to predict the rate of deterioration of patients who progress from mild or moderate disease to severe or critical disease. Eighty percent of patients will have asymptomatic or mild and moderate disease and will require no intervention.

Current South African guidelines only recommend paracetamol for the symptomatic treatment of this patient. Non-steroidal drugs used for clearly defined indications may be continued. The patient should also be encouraged to continue with his antihypertensive and anti-diabetic medication and, if possible, should monitor blood sugar more regularly. The patient presentation is one of the acute viral replication phases of the illness, and steroids are absolutely contraindicated as it can promote viral replication and accelerate the disease. Patients taking steroids during this phase of the illness showed worse clinical outcomes in the Randomised Evaluation of COVID-19 Therapy (RECOVERY) trial.

Lots of publicity on the use of vitamins and in some cases mega doses of vitamins have emerged in the last year. Some studies have shown better outcomes for severely ill, hospitalised patients who received Vitamin C and Vitamin D supplementation. An Italian study showed that up to 40% of hospitalised patients with COVID-19 were vitamin D deficient. The evidence for the use of zinc, vitamin C and vitamin D supplementation for patients with mild disease does not exist and should be a focus for future primary care studies.

Ivermectin has received immense publicity in South Africa, and many are using this product off-label. The World Health Organization and other national regulatory bodies around the world have not provided clear guidelines on the safety and efficacy of its use. Studies are ongoing and emerging evidence looks promising, but this is another area that requires a well-constructed trial in the primary care setting. At the time of writing, the South African Health Products Regulatory Authority has allowed for the compassionate use and access through the legal framework of Section 21 of the *Medicines and Related Substances Control Act* (101 of 1965 as amended).

### Further reading

- South African Department of Health and the National Institute of Communicable Disease. Clinical management of suspected or confirmed COVID-19 disease [homepage on the Internet]. 3rd ed. Pretoria. 2020 [cited 2021 Feb 01]. Available from: https://www.nicd.ac.za/wp-content/uploads/2020/08/Clinical-management-of-suspected-or-confirmed-COVID-19-V5-24-August-2020.pdf
- Li X, Liu C, Mao Z, et al. Predictive values of neutrophil-to-lymphocyte ratio on disease severity and mortality in COVID-19 patients: A systematic review and meta-analysis. Crit Care. 2020;24(1):1–10. https://doi.org/10.1186/s13054-020-03374-8

## Short answer question (SAQ): The family physician’s role in leadership and governance

You work as a family physician at a rural district hospital. As part of your responsibilities, you do a chart audit of patients seen in the outpatient’s department. A 75-year-old woman diagnosed with hypertension, hypothyroidism and ischaemic heart disease was seen last week. She was sent home with a month supply of the following medication:

- Carbamazepine 200 mg at night
- Tramadol 50 mg twice daily
- Amitriptyline 50 mg at night
- Wintergreen – apply topically as required
- Ibuprofen 400 mg three times a day
- Salbutamol 2 puffs as required
- Enalapril 10 mg twice daily
- Beclomethasone 1 puff daily
- Betamethasone cream – apply daily
- Furosemide 20 mg daily
- Amlodipine 5 mg daily
- Ramipril 5 mg daily
- Isosorbide dinitrate 10 mg twice daily
- Cinnarizine 15 mg twice daily
- Chlorphenamine 4 mg at night
- Atorvastatin 20 mg at night
- Thyroxine 0.05 mg daily.
  1. List four examples of irrational prescribing in this prescription. (4 marks)
  2. List four clinical questions that need to be addressed to rationalise the prescription at the next visit. (4 marks)
  3. If you saw this patient at her next visit, how would you go about resolving these four questions in terms of your history taking, side room tests or other investigations? (4 marks)
  4. A root cause analysis can identify the factors that might have contributed to the problems with this prescription. List four possible root causes and provide a brief explanation for each. (8 marks)

Total: 20 marks

Model answers

### 1. List four examples of irrational prescribing in this prescription. (4 marks)

(One mark to be awarded per reasonable answer from the list below)

1. Polypharmacy in the elderly increases the risk of harmful side effects.
2. High risk of harmful drug interactions and side effects. For example, chlorpheniramine and amitriptyline both cause sedation.
3. More than one drug is prescribed in same class; for example, angiotensin-converting enzyme inhibitor (ACE-I) is prescribed twice in different doses (ramipril and enalapril). Two antihistamines are prescribed (chlorpheniramine and cinnarizine).
4. More than one drug may be prescribed for the same condition unnecessarily; for example, two drugs are prescribed for possible neuropathic pain (carbamazepine and amitriptyline). Pain medications need to be rationalised.
5. Some of the medications are possibly detrimental to her existing medical conditions; for example, non-steroidal anti-inflammatory drugs (NSAIDs) are potentially harmful in elderly (presence of comorbid cardiovascular or renal disease or a increased risk of upper gastrointestinal bleeding) and contraindicated in asthma or hypertension.
6. Medications are prescribed for conditions not listed. Does she have these conditions: asthma, eczema, inner ear problems and pain condition?
7. Furosemide is a high-ceiling diuretic, and a current indication for furosemide is not clear; usually step down to hydrochlorothiazide unless contraindicated.
8. Other appropriate examples of irrational prescribing.

### 2. List four clinical questions that need to be addressed to rationalise the prescription at the next visit. (4 marks)

(One mark for each relevant clinical question)

1. Does she have asthma (to justify the short-acting beta-2-agonist and inhaled steroid)? If she does not have asthma, does she have some other condition causing shortness of breath such as cardiac failure, chronic obstructive pulmonary disease (COPD) or post-tuberculosis (TB) damage?
2. Does she have a dermatological condition (e.g. eczema or other condition requiring steroids)?
3. Does she have a history of atopy (e.g. rhinitis, eczema, asthma, urticaria) to justify chlorpheniramine?
4. Does she have a problem with chronic or neuropathic pain (to explain the carbamazepine, amitriptyline, tramadol, wintergreen, ibuprofen), or could she have epilepsy or depression or insomnia?
5. Does she have a problem with vertigo or inner ear disease, requiring cinnarizine?
6. Does she have hypercholesterolaemia, or does she require a statin for secondary prevention?
7. Does she have renal failure or chronic kidney disease?
8. Is her hypertension controlled?
9. Is her ischaemic heart disease (IHD) controlled?
10. Is her hypothyroidism controlled?
11. Other relevant questions, such as a mental health screen (exclude any sleep disorder or substance use disorder).

### 3. If you saw this patient at her next visit how would you go about resolving these four questions in terms of your history taking, side room tests or other investigations?

(One mark for a full answer to the question involving appropriate history, examination and side room tests or investigations)

1. Asthma or COPD or respiratory disease or cardiac failure:
  - History: Ask for a history of respiratory problems; contrasting features of atopy or asthma with COPD, history of TB, symptoms of cardiac failure.
  - Examination: Looking for signs of congestive cardiac failure (CCF), asthma, COPD or post-TB damage.
  - Tests: Chest x-ray to exclude cardiac failure and presence of respiratory disease; peak expiratory flow (PEF) and reversibility for asthma; and arrange echocardiogram at a level two hospital if indicated.
2. Dermatological condition:
  - History: Ask patient about skin disease and history of atopy.
  - Exam: examine for skin disease.
  - Tests: only needed if unclear.
3. Atopy:
  - History: Ask for the history of rhinitis, urticaria, eczema, asthma, etc.
  - Exam: examine according to the history of atopy.
4. Pain:
  - History: Ask patient about chronic pain problems, epilepsy and mental problems.
  - Exam: examine according to history.
5. Vertigo:
  - History: Ask for the history of vertigo or inner ear problems.
  - Exam: examine ear, nose and throat (ENT) system as required.
  - Ask: is this caused by the drugs?
6. Hypercholesterolaemia:
  - Check total cholesterol or lipogram if available.
7. Renal failure, chronic kidney disease (CKD):
  - Check urinalysis, creatinine, estimated glomerular filtration rate (eGFR) and electrolytes (K particularly).
8. Hypertension:
  - Check blood pressure; perform electrocardiogram (ECG).

### 4. A root cause analysis can identify the factors that might have contributed to the problems with this prescription. List four possible root causes and provide a brief explanation for each (8 marks)

(One point for the category of root cause analysis and one point for a satisfactory explanation)

1. Root cause analysis:
  - Clinical or cognitive factors: Poor clinical decision-making with underlying lack of knowledge on clinical pharmacology.
  - Individual or psychological: Poor performance/medical error because of depression, burnout, stress, personality or other issues affecting mental state or motivation.
  - Patient-related factors: Health literacy, communication barriers and patient demands.
  - Organisational-related factors: High clinician workload; poor supervision by responsible pharmacist and/or dysfunctional prescriber–dispenser relationship; insufficient senior support and mentorship of junior prescribers or dispensers; lack of system for review of prescribing practices (such as a pharmaceutical treatment committee); dysfunctional organisational culture with a lack of attention to accountability of individual clinicians and/or shared team responsibility (clinical governance and leadership).

#### Further reading

- National policy for patient safety incident reporting and learning in the public health sector of South Africa [homepage on the Internet]. Department of Health; 2016 [cited 2018 Sep 21]. Available from: https://www.idealclinic.org.za/docs/policies/National%20Policy%20for%20Patient%20Safety%20Incident%20Reporting%20and%20Learning%20in%20South%20Africa%207%20July%202016.pdf
- Promoting rational use of medicines [homepage on the Internet]. World Health Organization; 2002 [cited 2018 Sep 21]. Available from: http://www.who.int/medicines/publications/policyperspectives/ppm05en.pdf

## Critical appraisal of quantitative research

Read the accompanying article carefully and then answer the following questions (*total 30 marks*). As far as possible, use your own words. Do not copy text from the article. Be guided by the allocation of marks with respect to the length of your responses.

- Muchena G, Gombe N, Takundwa L, et al. Factors associated with contracting malaria in ward 29 of Shamva District, Zimbabwe, 2014. S Afr Med J. 2017;107(5):420–423. https://doi.org/10.7196/SAMJ.2017.v107i5.12204
  1. Does the title of the study relate to the aim of the study? Explain. (2 marks)
  2. The authors used an unmatched 1:1 case–control study design. Explain your understanding of this design. (3 marks)
  3. The researchers used a structured questionnaire to interview the participants. How could the authors have assured its validity? (4 marks)
  4. ‘The risk factors for contracting malaria were performing early morning chores (odds ratio [OR] 2.75; 95% confidence interval [CI]: 1.20–6.32)’. Please define and explain what you understand by this statement. (6 marks)
  5. Which risk factors demonstrated no association between the exposure and the disease? (2 marks)
  6. What do you understand by confounding, and how does it apply in this article? (3 marks)
  7. Using the acronym READER (Relevance, Education, Applicability, Discrimination, Evaluation and Reaction) analyse this article. (10 marks)

Model answers

### 1. Does the title of the study relate to the aim of the study? Explain. (2 marks)

The title (together with the abstract and keywords) is meant to motivate your reader to look at the full article. Typically, the title may describe the variables studied (independent and dependant), the study population, setting, design and time interval, as well as the main result/finding.

In this article, the aim of the study does relate to the title. Details included are the event of interest (contracting malaria), the study setting (Ward 29 of Shamva District, Zimbabwe) and the time interval (2014). However, the title does not describe the full scope of the study, as the study design and population are missing. A more complete title could have been: *Understanding the risk factors and health service response during a malaria outbreak in Ward 29 of Shamva District, Zimbabwe (2013/2014) – a retrospective case–control study.*

### 2. The authors used an unmatched 1:1 case–control study design. Explain your understanding of this design. (3 marks)

This is an observational study that compares groups of cases and controls. Study participants are selected for the study based on their outcome status. Cases are the participants who have the outcome of interest, whereas controls do not have the outcome of interest (in this study the outcome of interest was malaria according to a clear definition: signs and symptoms suggestive of malaria, together with a positive malaria rapid diagnostic test, diagnosed within the time frame of 23 December 2013 until 26 January 2014). Data relating to risk factors were collected retrospectively to determine differences in exposures between the cases and controls. Cases and controls are not allocated randomly to the exposure or intervention as in an experimental study. The differences in exposure levels between cases and controls serve as the basis for the estimation of association between exposure and the disease. The optimal case-to-control ratio is 1:1, as was used in this study. In some study designs, more controls than cases may have to be recruited if the study is limited by the number of cases available, to increase the statistical power.

### 3. The researchers used a structured questionnaire to interview the participants. How could the authors have assured its validity? (4 marks)

There are three main types of validity: content, face and construct validity.

Content and construct validity could be assured by use of an expert panel. Such a panel might consist of experts in research, as well as on the topic itself. This expert panel would address the question of whether the content of the survey is related to the research aim and that all relevant topics were included (and irrelevant excluded, [i.e. content]), and whether the questions were formulated in such a way that they are likely to provide an in-depth and comprehensive exploration of the topic (i.e. construct). (2 marks)

Following this piloting can address face validity: do the intended respondents find the questionnaire design to be relevant and meaningful, and are the questions clear and logical? (1 mark)

Piloting can also address practical issues of understanding, translation, time taken to complete the questionnaire and the best method of administering the questionnaire. (1 mark)

*Additional measure of validity (not part of model answer)*: Criterion validity addresses the correlation between the survey and a ‘gold standard’ measure of the same topic (if an existing tool is available that measures the same topic). In addition to measures of validity, measures of reliability (test–retest, inter-rater and internal consistency) should also be considered when developing questionnaires for survey research.

### 4. ‘The risk factors for contracting malaria were performing early morning chores (OR 2.75; 95% CI 1.20–6.32)’. Please define and explain what you understand by this statement. (6 marks)

Odds ratio (OR) is the odds of exposure in the diseased group divided by the odds of exposure in non-diseased group. It is used by epidemiologists in studies when looking for factors which do harm. It is a way of comparing patients who already have a certain condition (cases) with patients who do not (controls) as in a case–control study. Odds are calculated by the number of times an event happens by the number of times it does not happen.

Interpretation:

- If the diseased group has lower odds, the OR will be less than 1 (not linked to disease).
- If the non-diseased group has lower odds, the OR will be greater than 1 (exposure linked to disease).
- If there is no difference between the two groups, the OR will exactly be 1 (no association between the exposure and disease).

In the case of this study the odds ratio for performing early morning chores is greater than 1, and therefore the exposure is linked to the disease, and there is an increased risk of contracting malaria when performing early morning chores. (4 marks)

Because only a sample of the population can be measured, confidence intervals (precision) give a range in which you think the real answer lies with a given degree of certainty. Given the sample statistic (OR in this case) we can be 95% certain that the CI of 1.20–6.32 contains the true population parameter. In general, the larger the sample size, the smaller the CI, and vice versa. When the CI of a ratio crosses 1 (i.e. the range encompasses values showing increased and decreased risk), the statistical significance of the given ratio is weakened. (2 marks)

### 5. Which risk factors demonstrated no association between the exposure and the disease? (2 marks)

Staying in a sprayed home and staying indoors at night were deemed to be protective factors, as the OR was less than 1.

### 6. What do you understand by confounding and how does it apply in this article? (3 marks)

Confounders, also known as third variables, usually distort the relationship between an independent (exposure) and a dependent (outcome) variable. The distortion can then lead to erroneous conclusions in terms of cause and effect (association, correlation and causation). Statistical methods are available to adjust for potential confounding, including the regression models (stratified analysis and forward stepwise logistic regression analysis) used in this article.

### 7. Using the relevance, education, applicability, discrimination, evaluation and reaction, analyse this article (10 marks)

The READER format may be used to answer this question:

- Relevance to family medicine and primary care. (2 marks)
- Education: Does it challenge existing knowledge or thinking? (2 marks)
- Applicability: Are the results applicable to my practice? (2 marks)
- Discrimination: Is the study scientifically valid enough? (2 marks)
- Evaluation: From the above information, how would I score or evaluate the usefulness of the study to my practice? (1 mark)
- Reaction: What will I do with the study findings? (1 mark)

The answer may be a subjective response, but it should be one that demonstrates a reflection on the change, possible changes within the student’s practice within the Southern African health care system. The reflection on whether all important outcomes were considered is therefore dependent on the reader’s own perspective. (Is there other information you would have liked to see?)

A model answer could be written from the perspective of the family physician employed in the district health system:

This case–control study is relevant to the African primary care context, as the prevention, an early diagnosis and the treatment of malaria represent a key public health concern requiring a coordinated primary health care approach. The intended target audience is policymakers, health service managers and primary care teams, which makes the study findings relevant to the clinician at the coalface. The risk factors and health system preparedness and response may resonate with the challenges experienced in primary health care, as district teams need to respond in a collaborative manner to address the burden of malaria. In terms of discrimination, the study design of the case–control study appears to be sufficiently powered to infer key risk and protective factors for contracting malaria and make the conclusions drawn acceptable. The central role of a coordinated district health response was highlighted, especially regarding environmental and personal level factors. The study may be discussed with the local and district management team and used as basis for planning and tailoring the local health service response in endemic areas.

(Total: 30 marks)

### Further reading

- Mash B, Ogunbanjo GA. African primary care research: Quantitative analysis and presentation of results. Afr J Prim Health Care Fam Med. 2014;6(1):1–5. https://doi.org/10.4102/phcfm.v6i1.646
- Govender I, Mabuza LH, Ogunbanjo GA, Mash B. African primary care research: Performing surveys using questionnaires. Afr J Prim Health Care Fam Med. 2014;6(1):1–7. https://doi.org/10.4102/phcfm.v6i1.589
- Pather M. Evidence-based family medicine. In: Mash B, editor. Handbook of family medicine. 4th ed. Cape Town: Oxford University Press, 2017; p. 430–453.
- Naude C, Young T. How to search and critically appraise the literature. In: Goodyear-Smith F, Mash B, editors. How to do primary care research. 1st ed. Boca Raton, FL: CRC Press, 2019; p. 135–146.
- Stevens R. Statistics in primary care research. In: Goodyear-Smith F, Mash B, editors. How to do primary care research. 1st ed. Boca Raton, FL: CRC Press, 2019; p. 161–165.
- Ball L, Barnes K. How to conduct a survey in primary care. In: Goodyear-Smith F, Mash B, editors. How to do primary care research. 1st ed. Boca Raton, FL: CRC Press, 2019; p. 167–175.
- Joannabriggs.org. Critical appraisal tools [homepage on the Internet]. JBI; 2019 [cited 2021 Mar 03]. Available at: https://jbi.global/critical-appraisal-tools.
- CASP Checklists. Critical appraisal skills programme [homepage on the Internet]. c2018 [cited 2021 Mar 03]. Available from: https://casp-uk.net/casp-tools-checklists/
- MacAuley D. READER: An acronym to aid critical reading by general practitioners. Br J Gen Pract. 1994;44(379):83–85.

## Objectively-structured clinical examination (OSCE) scenario

### Objective of station

This station tests the candidate’s ability to apply rational investigations and prescribe for a patient with possible rheumatoid arthritis.

### Type of station

Integrated consultation.

### Role player

Middle-aged woman.

### Instruction to candidate

- You are the family physician working in the emergency unit at a district hospital.
- The following patient is known to the facility with hypertension. The nurse practitioner has referred her to you for a new problem.
- Please consult with this patient and develop a comprehensive plan.
- You do not need to perform a physical examination.
- Physical examination findings and investigations will be provided to you on request.

## Instructions for the examiner

### Objectives

This station tests the candidate’s ability to apply rational investigations and prescribe for a patient with possible rheumatoid arthritis.

This is an integrated consultation station in which the candidate has 14 min.

**FIGURE 1:**
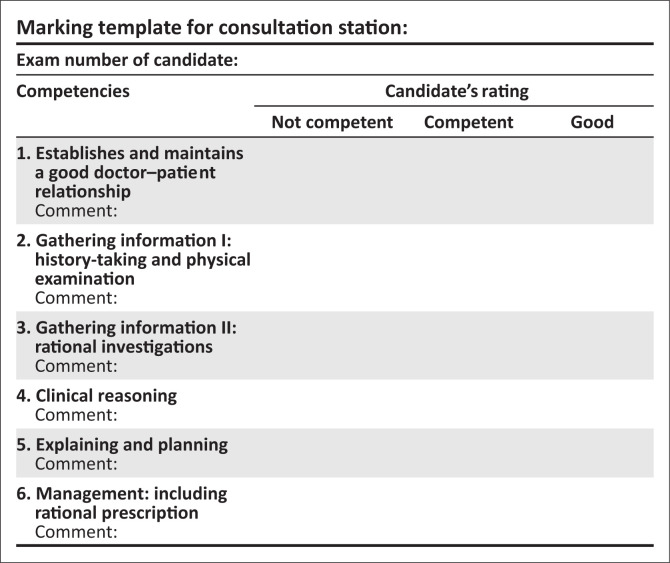
Marking template for consultation station.

- Familiarise yourself with the assessor guidelines (Figure 1), which detail the required responses expected from the candidate.
- No marks are allocated. In the mark sheet, tick off one of the three responses for each of the competencies listed. Make sure you are clear on what the criteria are for judging a candidates’ competence in each area.
- Provide the following information to the candidate when requested (delete if not applicable).
- Please switch off your cell phone.
- Please do not prompt the student.
- Please ensure that the station remains tidy and is reset between candidates.
- This station is 15 min long. The candidate has 14 min; then you have 1 min between candidates to complete the mark sheet and prepare the station.

### References

- South African Department of Health. Hospital Level Standard Treatment Guidelines and Essential Medicines List - Chapter 13.1. Pretoria: National Department of Health 2019.
- University of Cape Town, Division of Clinical Pharmacology. South African Medicines Formulary. 9th ed. 2010. Health and Medical Publishing Group. Cape Town. p. 386–397.

## Guidance for examiner

**Working definition of competent performance:** the candidate *effectively completes the task* within the allotted time, in a manner that *maintains patient safety*, even though the execution may not be efficient and well-structured.

1. Establishes a good doctor–patient relationship
  The *competent candidate* acts within the ethical framework (respects autonomy, justice, non-maleficence, beneficence). In addition, the *good candidate* displays empathy and compassion, acknowledging the patient’s discomfort and the anxiety related to ongoing physical symptoms.
2. Gathering information I: history and examination findings
  The *competent candidate* gathers sufficient information to identify current medical issues *(severe functional impairment; self-medicating with NSAIDs)* and any ongoing biopsychosocial risks. This candidate also assesses examination findings available at hand. In addition, the *good candidate* explores the patient’s experience, fears *(fear of disability),* expectations and health-seeking behaviour *(self-medicating)* and identifies opportunities for health promotion *(improved relationships; managing risks pro-actively).* This candidate requests examination findings for the key joints involved in rheumatoid arthritis (RA) *(shoulders, elbows, wrists, distal interphalangeal* [*DIP*] *and proximal interphalangeal* [*PIP*] *joints, knees)* and performs a thorough systemic examination *(cardiac, respiratory, abdominal, urine dipsticks).*
3. Gathering information II: rational investigations
  The *competent candidate* would identify the cost-effective, first-line diagnostic investigations *(x-ray both hands; need for C-reactive protein* [*CRP*], *erythrocyte sedimentation rate* [*ESR*], *rheumatoid factor* [*RF*]). In addition, the *good candidate* recommends holistic and comprehensive investigations to establish a baseline for future reference *(full blood count, alanine transaminase, serum uric acid).*
4. Clinical judgement
  The *competent candidate* uses available evidence to make the correct working diagnosis *(well-controlled hypertension* [*HPT*] *with mild renal impairment; possible rheumatoid arthritis)*. The *good candidate* can make a comprehensive, three-stage assessment *(as for ‘competent’ + inappropriate use of NSAIDs; fear of disability; impact on occupational function)*.
5. Explaining and planning
  The *competent candidate* clearly explains the working diagnosis *(no jargon; comprehensive; simple language)* and possible interventions. The *good candidate*, in addition, provides a platform for the patient to engage as an equal partner in sharing information and decision-making.
6. Management
  The *competent candidate* uses current evidence-based guidelines to develop a management plan *(pain management by using safe medications – short-term prednisone; need for disease-modifying anti-rheumatic drugs* [*DMARDS*]; *avoids NSAIDs; commits to long-term care; refers to Rheumatologist, or starts DMARDs)*. In addition, the *good candidate* develops a comprehensive plan by using the biopsychosocial approach *(as for ‘competent’ + patient advice on risk of disease progression and disability; offers assistance with occupational relief, mentions/refers to multidisciplinary team).*

## Examination findings and investigations

Add the relevant details for the examiner – examiner should NOT show all the examination findings to the candidate but should respond to specific questions being asked.

### General examination

- Blood pressure (BP) 150/95.
- Pulse 88/min – Regular; all pulses palpable.
- Weight: 78 kg; body mass index (BMI): 28.
- No peripheral oedema.
- Urine dipsticks: 1 + protein has been present for last few visits.

### Hands

- Swollen bilaterally: proximal and distal interphalangeal joints of most fingers.
- Tender joint margins and peri-articular soft tissue.
- Painful range of movement.

### Musculoskeletal

- Shoulders – normal.
- Elbows – normal.
- Wrists – bilateral: Painful flexion, extension and mildly tender to palpation.
- Knees – normal.

### Respiratory

- Good air entry bilaterally and adequate lung expansion.
- No inspiratory or expiratory crepitation.

### Cardiovascular (CVS)

- Jugular venous pressure – Normal.
- No bruits detected.
- Apex beat not displaced and morphologically normal.
- Normal heart sounds.

### Abdomen

- No abnormalities detected.

### Previous investigation results

- Creatinine: 134 g/L.
- eGFR: 48 mL/min.

## Role play – instructions for actor

### Appearance and behaviour

Middle-aged lady, well-groomed.

### Opening statement

‘Doctor, the nurse asked me to come to you. I have this problem with my hands … they are painful all the time’.

### History

- Open responses: Freely tell the doctor.
  ■ You have hypertension for the last 5 years and take your medications on time.
  ■ The pain in your hands is really troublesome and has affected your ability to work properly. The problem started about 6 months ago and seems to be getting worse. The pain comes and goes – some days no pain, and then some days it is bad and your fingers swell up.
- Closed responses: Only tell the doctor if asked
  ■ Fear: Your mother (died 3 years ago) had rheumatoid arthritis – You are worried that this is the same thing.
  ■ Concern and impact: will you become disabled? Already you cannot type at work when your hands are swollen – you work as a data capturer at an auditing firm.
  ■ The pain is quite bad – worse in the mornings for about an hour and then slowly gets better during the day as you start moving about.
  ■ Most days you take Voltaren (diclofenac) tablets because they help with the pain – you buy them over the counter.
  ■ Expectation: information, diagnosis and need for specialist.

### Social history

- Married, 2 children in high school.
- Husband for 18 years, works as a construction foreman – Only recently started working when lockdown rules changed.
- No real hobbies or sport – life is too busy.

## Data Availability

Data sharing is not applicable to this article as no new data were created or analysed in this study.

